# The Drives for Driving Simulation: A Scientometric Analysis and a Selective Review of Reviews on Simulated Driving Research

**DOI:** 10.3389/fpsyg.2020.00917

**Published:** 2020-05-27

**Authors:** Alessandro Oronzo Caffò, Luigi Tinella, Antonella Lopez, Giuseppina Spano, Ylenia Massaro, Andrea Lisi, Fabrizio Stasolla, Roberto Catanesi, Francesco Nardulli, Ignazio Grattagliano, Andrea Bosco

**Affiliations:** ^1^Dipartimento di Scienze della Formazione, Psicologia, Comunicazione, Università degli Studi di Bari Aldo Moro, Bari, Italy; ^2^Department of Agricultural and Environmental Science, Faculty of Agricultural Science, University of Bari Aldo Moro, Bari, Italy; ^3^Giustino Fortunato University, Benevento, Italy; ^4^Department of Interdisciplinary Medicine, School of Medicine, University of Bari Aldo Moro, Bari, Italy; ^5^Commissione Medica Locale Patenti Speciali, Azienda Sanitaria Locale, Bari, Italy

**Keywords:** driving simulation, driving simulator, fitness to drive, scientometric analysis, review

## Abstract

Driving behaviors and fitness to drive have been assessed over time using different tools: standardized neuropsychological, on-road and driving simulation testing. Nowadays, the great variability of topics related to driving simulation has elicited a high number of reviews. The present work aims to perform a scientometric analysis on driving simulation reviews and to propose a selective review of reviews focusing on relevant aspects related to validity and fidelity. A scientometric analysis of driving simulation reviews published from 1988 to 2019 was conducted. Bibliographic data from 298 reviews were extracted from Scopus and WoS. Performance analysis was conducted to investigate most prolific Countries, Journals, Institutes and Authors. A cluster analysis on authors’ keywords was performed to identify relevant associations between different research topics. Based on the reviews extracted from cluster analysis, a selective review of reviews was conducted to answer questions regarding validity, fidelity and critical issues. United States and Germany are the first two Countries for number of driving simulation reviews. United States is the leading Country with 5 Institutes in the top-ten. Top Authors wrote from 3 to 7 reviews each and belong to Institutes located in North America and Europe. Cluster analysis identified three clusters and eight keywords. The selective review of reviews showed a substantial agreement for supporting validity of driving simulation with respect to neuropsychological and on-road testing, while for fidelity with respect to real-world driving experience a blurred representation emerged. The most relevant critical issues were the a) lack of a common set of standards, b) phenomenon of simulation sickness, c) need for psychometric properties, lack of studies investigating d) predictive validity with respect to collision rates and e) ecological validity. Driving simulation represents a cross-cutting topic in scientific literature on driving, and there are several evidences for considering it as a valid alternative to neuropsychological and on-road testing. Further research efforts could be aimed at establishing a consensus statement for protocols assessing fitness to drive, in order to (a) use standardized systems, (b) compare systematically driving simulators with regard to their validity and fidelity, and (c) employ shared criteria for conducting studies in a given sub-topic.

## Introduction

Driving is a multifaceted activity involving cognitive and physical tasks. It requires the integration of visual and perceptual stimuli, information processing, decision making, vehicle control responses, motor programming and execution, and the capability of carefully responding to a dynamic environment (Heikkilä et al., 1998; Anstey et al., 2005; Classen et al., 2006; Ranchet et al., 2011).

In order to measure driving behavior and to assess fitness to drive, researchers have been using different assessment tools over time. The gold standard seems to be the on-road assessment of actual driving performance. This kind of evaluation is considered costly, stressful, and time-consuming; furthermore, it is very difficult to evaluate the driving performance in different conditions, such as in heavy traffic, at night, in various types of weather, or in dangerous circumstances (i.e., collision avoidance, obstacles on the road). Moreover, testers often experience anxiety and stress, and experimenters do not completely manage to control all variables, such as errors and violations (e.g., Brown and Ott, 2004; Kraft et al., 2010).

Neuropsychological evaluation by means of psychometric tests is also used to evaluate driving behavior as well as fitness to drive. The underlying assumption is that significant cognitive impairments should prevent safe operation of a motor vehicle (e.g., Kraft et al., 2010). The most widely appraised cognitive domains are visual perception (e.g., contrast sensitivity; Uc et al., 2006a; Worringham et al., 2006), visual attention (e.g., Uc et al., 2006a, 2007), visual and verbal memory (e.g., Heikkilä et al., 1998; Radford et al., 2004; Uc et al., 2007), information processing (e.g., Heikkilä et al., 1998; Worringham et al., 2006), motor dexterity (e.g., Radford et al., 2004; Grace et al., 2005), executive functioning (Stolwyk et al., 2006; Uc et al., 2006b), and visuospatial organization and planning (e.g., Grace et al., 2005; Uc et al., 2007). Neuropsychological tests’ performance can predict driving ability, but initial evidences suggested that neuropsychological screening batteries explained less than 70% of the variance in driving ability and correctly classified about 70% of participants (e.g., see Heikkilä et al., 1998; Radford et al., 2004; Worringham et al., 2006; Amick et al., 2007; Devos et al., 2007). More recently, Verster and Roth (2012) showed that psychometric test batteries predicted on-road test performance at only 33.4%, showing that combinations of basic neuropsychological/psychometric tests are not always good predictors of driving performance. The screening batteries considered most reliable, with sensitivity and specificity ranging between 61 and 94%, included the Trail Making Test (TMT), the Useful Field of View (UFOV), the Pelli–Robson contrast sensitivity test, and the Symbol Digit Modalities Test (SDMT) (e.g., Jacobs et al., 2017). All together, these findings compel researchers to shed further light on the role of neuropsychological tests in predicting fitness to drive.

The use of a driving simulator is another widespread method for assessing fitness to drive (Shechtman, 2010). It provides the opportunity to test many challenging/hazardous conditions or events that may not be presented during on-road testing in a standardized setting. Moreover, a lot of advantages contribute to make this approach a promising alternative to both neuropsychological and on-road testing for a safe assessment procedure as well as for cost cutting, time efficiency, and reliability (Lew et al., 2005; de Winter et al., 2009; Shechtman et al., 2009; Mayhew et al., 2011). Additionally, a large amount of data could be collected, capturing several variables and measures. On the other hand, the main limitations of driving simulation seem to be: (a) the difficulty to compare research findings adopting different driving simulators because of how parameters are collected and how driving simulator performance is quantified (e.g., Jacobs et al., 2017) and (b) sickness, dizziness, nausea, vomiting, and sweating associated with simulations (Brooks et al., 2010; Domeyer et al., 2013).

A considerable amount of literature on driving simulation has been produced since 1970. The rapid and continuous advancements of technology in the last 50 years have allowed for a massive development and employment of driving simulators. A recent bibliometric analysis (Guo et al., 2019) has explored the paths through which literature on simulated driving has evolved in the last 20 years. Authors filtered out 3,766 documents published from 1997 to 2016 and performed several bibliometric computations. The Countries which contributed and collaborated most in publishing studies on simulated driving were the United States followed by Germany and China. The most productive institutes were located in Netherlands and in the United States. The most recognized journals were in transportation and ergonomics, and the most productive authors were “J. D. Lee,” “D. L. Fisher,” “J. H. Kim,” and “K. A. Brookhuis.” A co-citation analysis was also performed showing different trends in topic over time—from early works on task-induced stress, drivers with neurological disorders, alertness and sleepiness, driving assistance systems, driver distraction in the first 10 years to the effect of drug use on driving behavior, the validity of driving simulators, and automated driving in more recent years.

Regarding the latter point highlighted by Guo et al. (2019), in a recent literature review, Wynne et al. (2019) pointed out the poor consistency among measures employed to assess the simulated performance and on-road driving. Several studies do not report all the employed measures to assess simulated driving and/or do not provide a direct comparison with measures assessing driving performance in the real world. Authors claimed that these results suggest the lack of a common research practice. Indeed, evidences of validity on one measure in one simulator do not mean that other measures may be equally valid in the same simulator, or that the same measures can be considered valid in other simulators. Furthermore, each setup is unique even when modeled on previously validated simulators and may be validated in light of those uniqueness (Pinto et al., 2008). Thus, simulated driving cannot be considered a universally valid measure of on-road driving performance (George, 2003; Wynne et al., 2019).

Similarly, a lot of studies have been devoted to investigating the predictive validity of cognitive performance measured with paper-and-pencil neuropsychological tests on simulated driving performance. Several reviews in the last 20 years aimed to summarize the results provided from primary studies putting together specific cognitive tasks or tests able to predict both simulated and real driving measures (Reger et al., 2004; Mathias and Lucas, 2009). Despite the above, there seems to be no clear evidence supporting the validity of driving simulation measures compared to neuropsychological testing ones in the assessment of fitness to drive (Marcotte and Scott, 2004; Mathias and Lucas, 2009).

The driving simulators are widespread employed in research in several disciplines and for different aims. Moreover, they are widely used to assess driving performance and driving behavior in several populations (Marcotte and Scott, 2004; Wynne et al., 2019). Evidences of validity on simulated driving measures observed in specific populations are not representative of all populations. This issue increases the controversy in literature regarding driving simulators’ validation due to differences between studies also in special populations (Shechtman, 2010), and thus concerns remain regarding their employability. Taken together, the evidences from primary studies provide a framework of puzzling and blurred results which may prevent generalizable conclusions about the validity of driving simulators with respect to neuropsychological testing and on-road performance.

This variability among primary studies has elicited a high number of secondary studies. In order to gain a comprehensive picture of secondary studies and a “state-of-the-art” snapshot of the domain, a scientometric analysis was conducted exclusively on driving simulation reviews. The choice to filter only secondary studies will allow to have a sort of *second-order analysis* on the topic as well as an overview on the different uses of driving simulators across research fields and several academic disciplines.

The present work has two aims: 1) to perform a scientometric analysis on the corpus of reviews on driving simulation studies conducted in the last 30 years, i.e., from January 1, 1988, to July 1, 2019, and 2) to propose a selective review of reviews of the main clusters emerged from the scientometric analysis, with a special focus on psychometric issues related to validity of driving simulators compared to standardized neuropsychological and on-road testing as well as to fidelity with respect to real-world driving experience. Reviews may provide an overview of primary studies on a certain topic, thus highlighting similarities and differences among the findings of the studies included. While contemplating the extensive variability of the results provided in primary studies for each review, the review of reviews is aimed at better understanding the effectiveness of driving simulator in predicting measures of fitness to drive, with respect to both neuropsychological and on-road testing.

The scientometric analysis and the review focused on secondary studies could summarize more clearly whether the driving simulator is a useful and effective tool for the assessment of the fitness to drive, specifying in which discipline or population this happens, in a reliable manner. A comprehensive overview given by secondary studies could be also useful in order to highlight critical issues related to the effectiveness of driving simulators.

## Scientometric Analysis

### Materials and Methods

#### Data Collection

The great variety of disciplines interested in the topic of driving simulation and the not perfect overlap in search results on scientific databases has required to proceed with a search on two databases, thus improving the likelihood to carry out a fully exhaustive work (e.g., Meneghini et al., 2006; Pollack and Adler, 2015). Consequently, a literature search was conducted on July 1, 2019, on two databases, Scopus and Web of Science (WoS). The former is the largest abstract and citation database of peer-reviewed research literature in the fields of science, technology, medicine, social sciences, and arts and humanities. The latter is composed of several citation indexes for different disciplines, from social sciences to engineering to chemical sciences *et cetera*.

The search expression used for data collection was “Driv^∗^ Simulat^∗^” OR “Simulat^∗^ Driv^∗^” in the “title, abstract, keywords” search in Scopus database and in “Topic” search in WoS, which comprises title, abstract, author, keywords, and Keywords Plus. Scopus search returned 15,518 records, and WoS search returned 10,379 records. Such results were refined selecting “Review” in the field “Document type” of each database. There were 228 documents classified as reviews in Scopus and 151 in WoS. Two datasets containing several information for each record, such as abstract and keywords, bibliographical information, citation information, funding details, and the list of references, were exported in BibTeX format. Subsequently, they were converted into *dataframes* using bibliometrix R package (Aria and Cuccurullo, 2017) and merged together. After deleting duplicates, the final sample was composed of 298 records.

#### Data Analysis

Bibliometrics, scientometrics, and infometrics are methodological and quantitative approaches in which the scientific literature itself becomes the subject of analysis. Although their historical origins differ and they are not necessarily synonymous (Hood and Wilson, 2001), nonetheless, they share theories, methods, technologies, and applications. Their main aim is to measure the evolution of a scientific domain, the impact of scholarly publications, and the process of scientific knowledge production, and they often comprehend the monitoring of research in a given field, the assessment of the scientific contribution of authors, journals, or specific articles, as well as the analysis of the dissemination process of scientific knowledge (Mao et al., 2015).

Several tools and software have been developed and proposed in order to perform scientometric analysis, among the most known there are BibExcel, Bibliometrix R Package, CiteSpace, VOSviewer, *et cetera*. For the present work, two of them were used, namely, Bibliometrix R Package (Aria and Cuccurullo, 2017) and VOSviewer (Van Eck et al., 2010). Bibliometrix R Package is an open-source tool for quantitative research in scientometrics and bibliometrics that includes all the main bibliometric methods of analysis. VOSviewer is an open-source software tool for constructing and visualizing, among other functionalities, bibliometric networks of relevant information extracted from a body of scientific literature.

For the present study, a particular focus has been given to performance analysis, i.e., the statistical analysis based on publication outputs and received citation to gauge the research performance and also the leadership of Institutes, Departments, Journal or Persons (Noyons et al., 1999; Van Raan, 2004; Cantos-Mateos et al., 2012; Muñoz-Écija et al., 2017; Vargas-Quesada et al., 2017). Performance of Countries, Journals, Institutes, and Authors which published reviews on simulated driving was analyzed in order to highlight research contents and trends associated with such topic. Cluster analysis based on authors Keywords Co-occurrence Network (KCN) was employed in order to conceptualize the deep structure of the research field and its trends throughout different disciplines and methodologies.

### Results

#### Performance of Countries

Table 1 shows the number of reviews on driving simulation studies by Country, the number of single and multiple Country publications (SCP and MCP, respectively), and the Relative International Collaboration Rate (RICR; Elango et al., 2015) for the 10 most productive Countries. It can be noted that those Countries have produced together almost the 70% of all the reviews, with a high prominence of the United States, followed by the most industrialized Countries all over the world. The number of SCP and MCP together with the RICR may provide a measure of the degree of collaboration between different Countries. Australia, Netherlands, Canada, United Kingdom, and China showed an international collaboration rate equal or greater than the global rate (= 1), while the other five Countries showed an international collaboration rate lesser than the global rate. Supplementary Table S1 contains the number of reviews, the number of SCP and MCP, and the RICR for all the Countries present in the dataframe.

**TABLE 1:** Number of reviews on driving simulation studies, single and multiple Country publications, and Relative International Collaboration Rate for the 10 most productive Countries.

**Country**	**Number of reviews**	**SCP**	**MCP**	**RICR**
United States	87	55	32	0.85
Germany	31	23	8	0.60
Canada	20	10	10	1.16
France	15	10	5	0.77
United Kingdom	14	7	7	1.16
Australia	12	1	11	2.13
Netherlands	8	3	5	1.45
China	7	4	3	0.99
Poland	6	4	2	0.77
Switzerland	6	4	2	0.77

*SCP, Single Country Publications; MCP, Multiple Country Publications; RICR, Relative International Collaboration Rate.*

**FIGURE 1:**
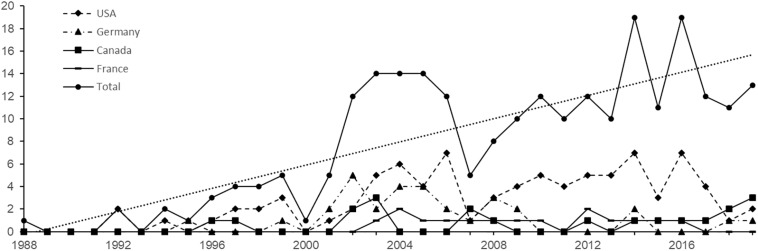
Number of reviews by Country and by year for the first four most productive Countries and total number of reviews by year from 1988 to 2019.

Figure 1 shows the number of reviews by Country and by year, from 1988 to 2019, for the first four most productive Countries, and the total number of reviews in the same years range. A visual inspection of the graph shows that the total trend is mimicked by that of the United States and only in part by that of Germany, which are the first two most productive Countries. It also emerges that after the year 2000, there has been a strong increase in the number of reviews, followed by a substantial drop in the year 2007 and a constant recovery in subsequent years, with high levels of interest in the last 5 years. As Guo et al. (2019) stated in their recent scientometric analysis on primary studies, the rapid advancement of technological tools applied to driving simulation in the last 20 years promoted thousands of studies and conversely a high interest for summarizing their findings.

#### Performance of Journals

Table 2 shows the number of reviews on driving simulation studies by the top 10 journals, as well as the total global citation score (TGCS), which refers to the number of times the document has been cited in the scientific databases used for retrieval. The number of citations was a piece of information present in the bibliographic record for each review, no matter where it came from (Scopus or WoS). The software we used for obtaining the performance of journals simply summed up the citations of the reviews published on each journal. The 10 most productive journals on a total of 223 journals accounted for 18.79% of the total 298 reviews. Surprisingly, it can be noted that only two journals in the top 10 belong to the transportation field (i.e., *Transportation Research Record* and *Traffic Injury Prevention*) with a relatively low citation score, while journals in the ergonomics and human factors field have a high citation score. In the first place *ex aequo* with another journal, there is a journal devoted to sleep medicine (i.e., *Sleep Medicine Reviews*) with a high number of global citations, as a demonstration of the strong interest for the relationship between sleep-related disorders and simulated driving. Two national journals (i.e., *VDI Berichte* and *Medycyna Pracy*) are also present, with a scarce number of citations. A decision was made not to exclude them in the retrieval phase in order to have a broader representation of the topic. Indeed, 258 reviews were published entirely in English language, and 40 were published in a double language, i.e., abstract in English and text in another language, or entirely in another language, nonetheless, all of them were indexed in Scopus or in WoS. Moreover, several journals with two (seven journals) or even only one (16 journals) review published obtained a good or very good performance, having more than 100 global citations. Supplementary Table S2 contains the number of reviews and the global number of citations for all the Countries present in the dataframe.

**TABLE 2:** Number of reviews on driving simulation studies by Source and total global citation scores.

**Source**	**Number of reviews**	**TGCS**
Sleep Medicine Reviews	12	637
VDI Berichte	12	9
Transportation Research Record	6	27
Human Factors	4	897
American Journal of Occupational Therapy	4	103
Traffic Injury Prevention	4	47
Medycyna Pracy	4	14
International Journal of RF and Microwave Computer-Aided Engineering	4	7
Ergonomics	3	214
Frontiers in Psychology	3	46

*TGCS, Total Global Citation Score.*

#### Performance of Institutes

Table 3 shows the number of reviews on driving simulation studies by the top 10 Institutes of the first author, by Country, as well as total global citation scores, and total citations per year. The most productive Institutes are located in the United States; University of Florida, Yale University, University of Iowa, University of Massachusetts, and University of Michigan have the highest global citations, as well as the highest total citations per year. Other productive Institutes are distributed worldwide between Europe, i.e., in Poland, Iceland and in the Netherlands, and Canada. Utrecht University and University of Toronto obtained a comparable high number of total citations and citations per year. Nofer Institute of Occupational Medicine and Reykjavik University obtained a lower number of total citations and citations per year. It is noteworthy that there are a number of Institutes which published one or two reviews that have reached a high or very high number of total citations and citations per year, such as Harvard University (United States, Number of reviews: 2, TGCS: 799, TCpY: 73.40), CNRS-Collège de France (France, Number of reviews: 2, TGCS: 546, TCpY: 41.27), University of Maryland (United States, Number of reviews: 2, TGCS: 395, TCpY: 59.48), Max Planck Institute (Germany, Number of reviews: 1, TGCS: 1,238, TCpY: 77.38), and University of Illinois (United States, Number of reviews: 1, TGCS: 583, TCpY: 53.00). Supplementary Table S3 contains the number of reviews by Institutes, by Country, as well as total global citation scores and total citations per year for all the Institutes present in the dataframe.

**TABLE 3:** Number of reviews on driving simulation studies by Institute, Country, total global citation scores, and total citations per year.

**Institute**	**Country**	**Number of reviews**	**TGCS**	**TCpY**
University of Florida	United States	8	200	23.95
Yale University	United States	5	307	13.69
University of Iowa	United States	5	147	13.80
Nofer Institute of Occupational Medicine	Poland	5	23	4.11
Utrecht University	Netherlands	4	165	11.97
Reykjavik University	Iceland	4	7	1.71
University of Massachusetts	United States	3	129	9.84
University of Toronto	Canada	3	112	8.58
University of Western Ontario	Canada	3	80	5.00
University of Michigan	United States	3	43	4.50

*TGCS, Total Global Citation Score; TCpY, Total Citations per Year.*

#### Performance of Authors

Table 4 shows the number of reviews on driving simulation studies by the top 10 Authors and the number of single-, multi-, and first-authored reviews for each Author. These Authors wrote or co-wrote 37 out of 298 (i.e., about 12.4%) reviews on driving simulation. “S. Classen” dominates the ranking with seven reviews, followed by “D.L. Fisher,” “S. Koziel,” and “M. Rizzo” with four reviews each, and all other Authors wrote three reviews each. Only five reviews were single-authored, and about half of them (19) were first-authored by one of the top 10 Authors.

**TABLE 4:** Number of reviews and number of single-, multi-, and first-authored reviews on driving simulation studies by Author.

**Author**	**Number of reviews**	**Single-Authored**	**Multi-Authored**	**First-Authored**
Classen S.	7	0	7	4
Fisher D.L.	4	0	4	1
Koziel S.	4	1	3	2
Rizzo M.	4	1	3	2
Andysz A.	3	0	3	2
Bekasiewicz A.	3	0	3	1
George C.F.P.	3	3	0	3
Pearlson G.D.	3	0	3	0
Uc E.Y.	3	0	3	1
Verster J.C.	3	0	3	3

#### Cluster Analysis

**FIGURE 2:**
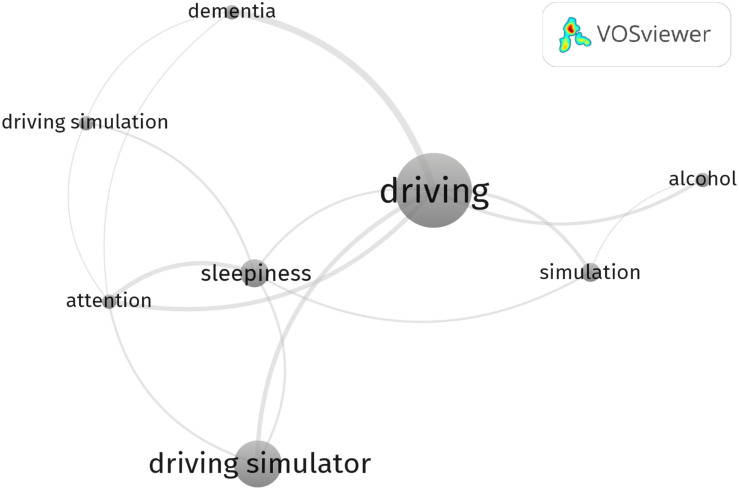
Author’s keywords network as a result of cluster analysis.

To identify and understand possible ensembles of semantic knowledge in this scientific area, a cluster analysis based on the KCN was performed. Cluster analysis is a multivariate technique that allows to minimize the distance between items belonging to the same group and to maximize the distance between items from different groups (Irani et al., 2016). VOSviewer software perform a cluster analysis throughout the “VOS mapping technique,” which is based on a weighted and parameterized variant of modularity-based clustering (for a detailed explanation, see Waltman et al., 2010). Keyword co-occurrences refer to the common presence, frequency, and proximity of keywords that are similar to others, i.e., based on the same topic, but not exactly the same. In other words, keyword co-occurrence is an association or combination of terms that marks the presence of a keyword in several papers (more than one) of a bibliographic database. Since the keywords of a paper are supposed to indicate the core concept of the study, this method is useful to systematically explore the knowledge-components and the knowledge-structure constructed by the keywords of papers in a specific research field. The KCN’s modularity is the network ability to decompose into separated modules or clusters. Each link between keywords in the network has a strength represented by a positive numerical value; the higher this strength value, the stronger the linkage (Radhakrishnan et al., 2017). The total link strength represents the number of publications in which two keywords occur together. In other words, link strength refers to the strength of semantics association between keywords. Highly cited keywords were analyzed and visualized with VOSviewer (Van Eck and Waltman, 2014). The type of analysis was selected by choosing “Co-occurrence” among the alternatives offered by the software. Subsequently, the analysis’ unit was chosen selecting only the “author’s keywords” and excluding “keywords plus” in which there were general and non-specific terms such as “human,” “review,” and “computer.” Furthermore, the counting method employed in this analysis was the “Fractional counting,” in which the weight of a link is fractionalized. For example, if a keyword co-occurs in a document with five other keywords, each of the five co-occurrences has a weight of 1/5. Considering the minimum and the maximum number of possible co-occurrences in the database (respectively 1 and 13), the co-occurrences threshold (i.e., the minimum number of occurrences of a keyword to enter the network) was based on the median value and set as 7. In this way, only eight of the 763 keywords in the database met the threshold and were brought into visualization (Figure 2). The purpose of this choice was to extract and visualize only the most relevant keywords. According to VOSviewer manual, the nodes represent the keywords, and the co-occurring frequency of a keyword is represented by the circle size; the larger a circle, the more a keyword has been co-selected in the driving simulation reviews. The analysis clearly defined three clusters: cluster 1 includes “driving simulator,” “driving simulation,” “sleepiness,” and “attention” grouping together ergonomic, anthropic, environmental, and psychophysiological factors; cluster 2 includes “driving,” “simulation,” and “alcohol” grouping very different human, environmental, and technical subtopics linked to driving simulation research; finally, cluster 3 includes only “dementia” which refers to a wide range of subtopics (i.e., assessment; treatment; assistive driving systems, etc.). Table 5 lists the main clusters identified, as well as the associated keywords, the occurrences, the links, and total link strength. Keyword “Sleepiness” was the most cited of cluster 1 and had the highest number of occurrences (13), links (five), and a total link strength equal to 8. Keyword “Attention” had seven occurrences, five links, and a total link strength of 7. Keyword “Dementia” had the same number of occurrences of “Attention,” but a fewer number of links (three) and a lower total link strength (5). Keyword “Driving simulation” also had the same number of occurrences, and a number of links equal to 3 and total link strength equal to 3. Keyword “Driving” was the most cited of cluster 2 and of the whole network, had the highest number of occurrences (35) and links (six) with a total link strength equal to 14, suggesting for a key role in the network. Keyword “Simulation” had 22 occurrences, three links, and achieved a total link strength of 6. Keyword “Alcohol” had seven occurrences, two links, and a total link strength of 3. Keyword “Driving simulator” was the only one present in cluster 3, with 22 occurrences, three links, and a total link strength of 5.

**TABLE 5:** Keywords and related occurences, links, and total link strength for the three major clusters.

**# Cluster**	**Keyword**	**Occurrences**	**Links**	**Total link strength**
1	*Sleepiness*	13	5	8
1	*Attention*	7	5	7
1	*Dementia*	7	3	5
1	*Driving simulation*	7	3	3
2	*Driving*	35	6	14
2	*Simulation*	9	3	6
2	*Alcohol*	7	2	3
3	*Driving simulator*	22	3	5

### Discussion

The first purpose of the present study was to perform a scientometric analysis on driving simulation reviews and to provide a comprehensive picture of secondary studies on this topic based on 298 reviews obtained by Scopus and WoS Core Collection in the last 30 years.

Performance analysis was conducted on Countries, Journals, Institutions, and Authors. The United States and Germany are the first two Countries for the number of driving simulation reviews, and their production has increased constantly in the last 20 years. Surprisingly, journals which contributed to the highest number of driving simulation reviews comprise only two titles belonging to the transportation field. This could be taken as a cue of the wide interest in the topic from different disciplines, such as medicine, engineering, psychology, *et cetera*. Regarding performance of institutes, again, the United States is the leading Country with five Institutes in the top 10. Top authors wrote from three to seven reviews each and belong to institutes located in North America (United States and Canada) and Europe (Netherlands, Germany, Poland, and Iceland).

The comparison between the results of the present study on the reviews of simulated driving and those obtained by Guo et al. (2019) on primary studies allows some considerations. Regarding performance of Countries, it is noteworthy that the United States and Germany dominate both ranks. Although Canada did not appear in the rank of the first four most productive Countries for primary studies, it appears as the third most productive Country in the rank of the reviews. Several European Countries, namely, France, United Kingdom, Netherlands, Poland, and Switzerland, are present in the top 10 of the reviews. Finally, even though in 2016, China was the second most prolific Country of primary studies in the world, in the reviews’ rank, it is located at the seventh position. This is an interesting result because it shows an increase in the gap between the United States and China switching from primary studies to reviews or a clear preference of China research centers for empirical studies.

The comparison between the performance of journals shows that those present in both ranks refer to the fields of transportation and engineering on one hand and to the field of human factors and ergonomics on the other. More specifically, Transportation Research Record, Human Factors, and Traffic Injury Prevention are classified, respectively, at the third, fourth, and sixth positions in both ranks. Two national journals are in the top 10 of the reviews, namely, *VDI Berichte* (Germany) and *Medycyna Pracy* (Czechia). This seems to be a clue of the attention European Countries devotes in reviewing and summarizing studies on driving simulation. Furthermore, Guo et al. (2019) emphasized that several journals published primary studies on the topic of sleepiness, but none of them appeared in the top 10 list. Regarding the reviews, it is noticeable that *Sleep Medicine Reviews* is in the first position. This highlights that sleepiness is one of the most recurrent topics in both primary and secondary studies on driving simulation. Indeed, sleepiness leads to physical conditions which may increase the rate of accidents and reduce the safety during driving (Guo et al., 2019). A comparison of citation scores among journals belonging to different subjects and disciplines is not desirable in this case, since differences in such scores may be partly due to differences in status and spread of journals themselves (e.g., impact factor and other bibliographic indexes related to the journals). Nonetheless, it is reasonable to think that all the reviews as well as the most part of the citations refer to the same topic, and that driving simulation is a topic which cannot be ascribed to one subject in particular, but belongs to several disciplines and research fields.

Regarding the performance of institutes, three Universities in the United States (i.e., University of Iowa, University of Massachusetts, and University of Michigan) are present in both the top 10 ranks. European institutes are almost equally present in the primary studies rank than in the reviews’ rank. Utrecht University is currently in the top 10 reviews’ rank, while Delft University of Technology and University of Groningen are among the most productive institutes regarding primary studies. Further, the Nofer Institute is located at the fourth position in the reviews’ rank. Iceland and Canada are represented by Reykjavik University and University of Toronto and University of Western Ontario, respectively. These institutes were not present in the top 10 rank of primary studies.

Concerning the most prolific authors, “D.L. Fisher” is the only author who appears to be present in both top 10 ranks. No other correspondences emerge by the comparison of authors’ performances among the two top 10 ranks.

The conceptual structure of driving simulation reviews was outlined using a co-occurrence network analysis to map and cluster high-frequency author keywords. Cluster analysis makes clear the interdisciplinary nature of this research topic. Three main clusters were identified together with eight relevant keywords. It is noteworthy that the eight keywords represent two distinct areas of interest, namely, an area devoted to the investigation of technical factors of driving simulation and another area devoted to the investigation of human factors, taking into account participants coming from special populations (i.e., persons with dementia, sleep-related disorders, alcohol-related disorders, and attention deficit). The choice to set a co-occurrences threshold using the median value among those available probably led to the best trade-off between the high heterogeneity of research topics and the need to summarize the main trends within the driving simulation framework.

A direct comparison between the results of cluster analysis proposed in this study and those from the cluster analysis conducted by Guo et al. (2019) is not possible. Indeed, in the present study, the cluster analysis was conducted on the Co-occurrence Network between authors’ keywords. In the study conducted by Guo et al. (2019), the cluster analysis was based on the Co-citation Network. The different nature of the data allows only a tentative comparison of semantic labels emerged by the respective cluster analyses. Labels associated with the human factor were predominant in both primary (10/13; 76.2%) and secondary studies (5/8; 62.5%). These labels were in the top positions in terms of productivity (number of documents) in the analysis on primary studies and were also present as authors’ keywords in a large amount of the reviews. This may be an indirect clue that simulated driving is a topic still strongly related to the human component. For example, driving simulation methods are employed to assess human driving abilities in different medical conditions, under the effect of various substances and medications, and to study safety behaviors and cognitive functioning related to driving activity.

## Selective Review of Reviews

### Methods

Cluster analysis based on authors’ keywords identified a total of 61 reviews. In order to identify the most relevant results about validity and fidelity of driving simulation, it was decided to conduct a further investigation within this subgroup. A checklist was created in order to answer for each review to the following questions: (1) Was driving simulation performance associated with or predictive of on-road testing performance? (2) Was driving simulation performance associated with or predictive of standard neuropsychological testing performance? (3) Did driving simulation exhibit the same or similar features of real driving? (4) Was a formal meta-analysis feasible? (5) Was there any critical issue highlighted regarding driving simulation?

The first two questions were aimed at investigating validity of driving simulations with respect to the other two methods currently used for assessing fitness to drive, i.e., on-road testing and standardized neuropsychological testing. The third question was aimed at investigating fidelity about the experience of driving simulation with respect to the experience of real driving. The fourth question was aimed at investigating the possibility of summarizing throughout meta-analytic techniques quantitative results for a given research topic. The fifth question was aimed at investigating critical issues regarding the use of driving simulation in research and clinical practice.

The answers to the first two questions were coded as following: “Yes” if in the text of the review Authors clearly stated that there was an association or a prediction between driving simulation and on-road and standardized neuropsychological testing, respectively; “No” if Authors clearly stated that there was no association or a prediction between driving simulation and on-road and standardized neuropsychological testing, respectively; and “Mixed results” if Authors stated that a low association or prediction was found or alternatively that an association or prediction was found for a subgroup of studies included in the review but not for others, “nd” if it was not possible to detect information about the relationship between driving simulation and on-road and standardized neuropsychological testing. The answers to the third question were coded as following: “Yes” if in the text of the review, Authors clearly stated that driving simulation had the same or similar features of real driving; “No” if Authors clearly stated that driving simulation had not the same or similar features of real driving or that had different and not comparable features; and “Mixed results” if Authors stated that driving simulation had only few features comparable to those of real driving, “nd” if it was not possible to detect information about the features shared between driving simulation and real driving. The answers to the fourth question were coded as following: “1” if a critical or narrative or clinical or selective review was conducted, “2” if a systematic review was conducted following international well-established guidelines for collecting data and reporting results, such as PRISMA Statement, CONSORT Statement, QUOROM guidelines, *et cetera*, “3” if a formal meta-analysis was conducted, i.e., it was possible to obtain a pooled effect size starting from the effect sizes of primary studies and to perform publication bias as well as moderator and sensitivity analyses. In order to answer the fifth and last questions, it was decided to extract from each review the sentences highlighting critical aspects and issues specifically linked to the use of driving simulation within the covered topic (see Supplementary Table S4).

### Results

Table 6 reports information obtained through the first four questions proposed. For each review, the following information was included in the table: cluster and keyword to which the review belongs to, the title and the reference of the review, the topic covered by the review, the discipline of the first Author, the answer to the first four questions proposed. Regarding the first question, i.e., the validity of driving simulation compared to the on-road testing, 36 out of 61 reviews reported an answer: 22 reviews reported an association between or a prediction of driving simulation with respect to on-road testing, seven did not report such an association or a prediction, and seven reported mixed results. Regarding the second question, i.e., the validity of driving simulation compared to standardized neuropsychological testing, it was possible to retrieve an answer for 24 out of 61 reviews: 21 reviews reported an association between or a prediction of driving simulation with respect to standardized neuropsychological testing, none of the reviews reported no association or prediction, while three reported mixed results. With respect to the third question, i.e., the fidelity about the experience of driving simulation with respect to the experience of real driving, 12 reviews out of 61 reported an answer: five reviews reported a comparable experience between driving simulation and real driving, three reported a non-comparable experience, and four reported mixed results. Concerning the fourth question, i.e., whether a formal meta-analysis was feasible, 47 reviews did perform a critical or narrative or clinical or selective review, 11 conducted a systematic review referring to well-established guidelines, and three were able to perform a formal meta-analysis and obtained a pooled effect size. With respect to the fifth question, i.e., what were the critical issues regarding driving simulation, several issues were reported (see Supplementary Table S4 for the full list of critical issues regarding driving simulation for each review), and the five most frequent ones were: (a) the lack of a common set of standards in order to reduce the variability of results between different types of simulators, (b) the phenomenon of simulation sickness, (c) the need for psychometric properties and normative data for both different parameters and specific populations, (d) the lack of studies investigating predictive validity of driving simulation with respect to crash and collision rates, and (e) the lack of studies investigating ecological validity of driving simulation in predicting real-world driving performance.

**TABLE 6:** Featuresof the studies included in the selective review of reviews.

**Cluster**	**Keyword**	**Title**	**Study**	**Topic**	**Discipline**	**Validity compared to on-road testing**	**Validity compared to laboratory testing**	**Fidelity**	**Systematic review**
1	Attention	Driving and neurologic disorders	Drazkowski and Sirven, 2011	Neurologic Disorders	Neurology	nd	nd	nd	1
1	Attention	Parkinson disease and driving: An evidence-based review	Crizzle et al., 2012	Major Neurocognitive disorders	Health Science	nd	Yes	nd	1
1	Attention	Neural correlates of simulated driving while performing a secondary task: A review	Palmiero et al., 2019	Distraction fMRI	Health Science	nd	nd	nd	1
1	Dementia	Driving and dementia: A review of the literature	Lloyd et al., 2001	Major Neurocognitive disorders	Occupational Therapy	No	nd	nd	1
1	Dementia	Driving and dementia: A review of the literature	Brown and Ott, 2004	Major Neurocognitive disorders	Psychiatry	Yes	Yes	nd	1
1	Dementia	Systematic review of driving risk and the efficacy of compensatory strategies in persons with dementia	Man-Son-Hing et al., 2007	Major Neurocognitive disorders	Geriatry	Yes	nd	nd	1
1	Dementia	Brain morphometry and functional imaging techniques in dementia: methods, findings and relevance in forensic neurology	Klöppel, 2009	Major Neurocognitive disorders	Psychiatry	nd	Yes	nd	1
1	Dementia	Car drivers with dementia: different complications due to different etiologies?	Piersma et al., 2016	Major Neurocognitive disorders	Psychology	Yes	Yes	nd	1
1	Driving simulation	Validation of driving simulators	Davison et al., 2011	Vision	Ophthalmology	nd	nd	nd	1
1	Driving simulation	Saccadic velocity as an arousal index in naturalistic tasks	Di Stasi et al., 2013a	Workload/Fatigue	Psychology	nd	nd	nd	1
1	Driving simulation	Inside the clinical evaluation of sleepiness: Subjective and objective tools	Baiardi and Mondini, 2019	Sleepiness	Medicine	Yes	Yes	nd	1
1	Driving simulation	Driving status of patients with generalized spike–wave on EEG but no clinical seizures	Antwi et al., 2019	Epilepsy and Driving	Neurology	nd	nd	nd	1
1	Sleepiness	Neuropsychological function in obstructive sleep apnea	Engleman and Joffe, 1999	Sleepiness	Medicine	Yes	Yes	nd	1
1	Sleepiness	Daytime sleepiness and its evaluation	Cluydts et al., 2002	Sleepiness	Psychology	No	nd	nd	1
1	Sleepiness	Cognition and daytime functioning in sleep-related breathing disorders	Jackson et al., 2011	Sleepiness	Health Science	nd	nd	nd	1
1	Sleepiness	Diagnostic approach to sleep-disordered breathing	Thurnheer, 2011	Sleepiness	Pneumology	nd	nd	nd	1
1	Sleepiness	Hypersomnolence and traffic safety	Gupta et al., 2017	Sleepiness	Psychiatry	nd	nd	nd	1
1	Sleepiness	Subjective and objective assessment of hypersomnia	Murray, 2017	Sleepiness	Neurology	nd	Yes	nd	1
1	Sleepiness	Determinants of policy decisions for non-commercial drivers with OSA: An integrative review	Rizzo et al., 2018	Sleepiness	Medicine	nd	nd	nd	1
1	Sleepiness	Driving simulators in the clinical assessment of fitness to drive in sleepy individuals: A systematic review	Schreier et al., 2018	Sleepiness	Neurology	Mixed results	nd	nd	2
1	Sleepiness	Narrative review: Do spontaneous eye blink parameters provide a useful assessment of state drowsiness?	Cori et al., 2019	Sleepiness	Medicine	Yes	Yes	nd	1
2	Alcohol	Using virtual reality to study alcohol intoxication effects on the neural correlates of simulated driving	Calhoun et al., 2005	Alcohol consumption	Psychiatry	Yes	nd	nd	1
2	Alcohol	A selective review of simulated driving studies: Combining naturalistic and hybrid paradigms, analysis approaches, and future directions	Calhoun and Pearlson, 2012	Neuroimaging and Alcohol	Psychology	Yes	nd	Yes	1
2	Alcohol	The sensitivity of laboratory tests assessing driving related skills todose-related impairment of alcohol: A literature review	Jongen et al., 2016	Alcohol	Pharmacology	No	nd	nd	2
2	Alcohol	A systematic review of the evidence for acute tolerance to alcohol – the “Mellanby effect”	Holland and Ferner, 2017	Alcohol	Medicine	nd	Yes	nd	2
2	Alohol	Effects of acute alcohol consumption on measures of simulated driving: A systematic review and meta-analysis	Irwin et al., 2017	Alcohol consumption	Health Science	Yes	nd	nd	3
2	Driving	Cognitive dysfunction in sleep disorders	Fulda and Schulz, 2001	Sleepiness	Psychology	Mixed results	Mixed results	nd	1
2	Driving	Outcome measurement in sleep medicine practice and research. Part 2: assessment of neurobehavioral performance and mood	Weaver, 2001	Sleepiness	Nursing	nd	Yes	nd	1
2	Driving	Are opioid-dependent/tolerant patients impaired in driving-related skills? A structured evidence-based review	Fishbain et al., 2003	Medication assumption	Psychiatry	nd	nd	nd	1
2	Driving	Driving simulators in clinical practice	George, 2003	Sleepiness	Medicine	No	nd	Yes	1
2	Driving	Residual effects of sleep medication on driving ability	Verster et al., 2004	Medication assumption	Pharmacology	No	nd	nd	1
2	Driving	The assessment of driving abilities	Marcotte and Scott, 2004	Assessment of driving skills	Psychiatry	Yes	Mixed results	No	1
2	Driving	Conversation effects on neural mechanisms underlying reaction time to visual events while viewing a driving scene: fMRI analysis and asynchrony model	Hsieh et al., 2009	Distraction fMRI	Communication science	nd	nd	nd	1
2	Driving	Functional consequences of HIV-associated neuropsychological impairment	Gorman et al., 2009	Major Neurocognitive disorders(hiv)	Psychiatry	Yes	Yes	Mixed results	1
2	Driving	Driving ability in Parkinson’s disease: Current status of research	Klimkeit et al., 2009	Major Neurocognitive Disorders	Psychology	Yes	Yes	nd	1
2	Driving	Phoning while driving II: A review of driving conditions influence	Collet et al., 2010	Distraction	Psychology	Mixed results	nd	Mixed results	1
2	Driving	A review of driving simulator parameters relevant to the operation enduring freedom/operation Iraqi freedom veteran population	Kraft et al., 2010	Iraqi Veterans/DS’s parameters operation	Medicine	Yes	Yes	yes	1
2	Driving	Zopiclone as positive control in studies examining the residual effects of hypnotic drugs on driving ability	Verster et al., 2011	Medication assumption	Pharmacology	Yes	Yes	nd	3
2	Driving	Systematic review of the quality and generalizability of studies on the effects of opioids on driving and cognitive/psychomotor performance	Mailis-Gagnon et al., 2012	Medication assumption	Medicine	nd	Mixed results	No	2
2	Driving	Does personality predict driving performance in middle and older age? An evidence-based literature review	Nichols et al., 2012	Personality	Psychology	No	nd	nd	2
2	Driving	Epilepsy and driving: Potential impact of transient impaired consciousness	Chen et al., 2014	Epilepsy	Neurology	Yes	nd	nd	1
2	Driving	Saccadic peak velocity as an alternative index of operator attention: A short review	Di Stasi et al., 2013b	Saccadic velocity/attention	Psychology	nd	nd	nd	1
2	Driving	The impact of depression on driver performance	Wickens et al., 2014	Mental health	Health Science	Yes	nd	nd	1
2	Driving	Driving in Parkinson’s disease	Özdilek and Uç, 2014	Major Neurodegenerative disorders	Neurology	nd	Yes	nd	1
2	Driving	The racer’s brain – How domain expertise is reflected in the neural substrates of driving	Lappi, 2015	Neural substrates of driving	Psychology	nd	nd	nd	2
2	Driving	Mirtazapine as positive control drug in studies examining the effects of antidepressants on driving ability	Verster et al., 2015	Medication assumption	Pharmacology	nd	nd	nd	1
2	Driving	High risk driving in treated and untreated youth with attention deficit hyperactivity disorder: Public health implications	Jillani and Kaminer, 2016	ADHD	Medicine	nd	nd	nd	2
2	Driving	Driving with a neurodegenerative disorder: An overview of the current literature	Jacobs et al., 2017	Major Neurocognitive Disorders	Neurology	Yes	Yes	yes	1
2	Driving	Covert hepatic encephalopathy: Can my patient drive?	Shaw and Bajaj, 2017	Hepatic Encephalopathy	Gastroenterology	nd	Yes	nd	1
2	Driving	Smart in-vehicle technologies and older drivers: A scoping review	Classen et al., 2019	Aging	Occupational Therapy	No	nd	nd	2
2	Driving	Relationships between cognitive functions and driving behavior in Parkinson’s disease	Ranchet et al., 2012	Cognition and driving in Parkinson	Psychology	Yes	Yes	nd	1
3	Driving simulator	The development of driving simulators: toward a multisensory solution	Pinto et al., 2008	Development of driving simulator	Psychology	Mixed results	nd	Mixed results	1
3	Driving simulator	Rehabilitation of combat-returnees with traumatic brain injury	Lew et al., 2009	Rehabilitation	Medicine	Yes	Yes	Yes	1
3	Driving simulator	Validation of driving simulators	Shechtman, 2010	Validation of driving simulators	Occupational Therapy	Mixed results	nd	Mixed results	1
3	Driving simulator	Nasal continuous positive airway pressure (nCPAP) treatment for obstructive sleep apnea, road traffic accidents and driving simulator performance: A meta-analysis	Antonopoulos et al., 2011	Effect of nasal continuous positive airway pressure	Medicine	Yes	nd	nd	3
3	Driving simulator	Effects of adaptive cruise control and highly automated driving on workload and situation awareness: A review of the empirical evidence	de Winter et al., 2014	Automated driving systems	Engineering	nd	nd	nd	2
3	Driving simulator	Establishing an evidence-base framework for driving rehabilitation in Parkinson’s disease: A systematic review of on-road driving studies	Devos et al., 2015	Major Neurocognitive disorders	Health Science	Yes	Yes	nd	2
3	Driving simulator	The impact of therapeutic opioid agonists on driving-related psychomotor skills assessed by a driving simulator or an onroad driving task: A systematic review	Ferreira et al., 2018	Medication assumption	Medicine	Yes	Yes	nd	2
3	Driving simulator	Evaluation method regarding the effect of psychotropic drugs on driving performance: A literature review	Iwata et al., 2018	Drugs consumption	Psychiatry	Mixed results	nd	No	1
3	Driving simulator	Efficacy of training with driving simulators in improving safety in young novice or learner drivers: A systematic review	Martín-delosReyes et al., 2019	Training for improving driving safety	Medicine	Mixed results	nd	nd	1
3	Driving simulator	Bibliometric analysis of simulated driving research from 1997 to 2016	Guo et al., 2019	Bibliometric analysis	Business	nd	nd	nd	1

### Discussion

As shown in Table 6, questions mainly represented in the reviews analyzed concerned the validity of driving simulation for the assessment of fitness to drive with respect to on-road and standardized neuropsychological testing.

Regarding the first question, it is noteworthy that a considerable effort has been done in order to demonstrate the validity of driving simulation techniques to those coming from ecological settings, such as on-road testing. Most of the reviews which gave a response about this question stated that it is possible to claim a significant association or prediction of driving simulation performance with respect to on-road testing performance. Nevertheless, recurring critical issues related to this question emerged. Firstly, psychometric properties of driving simulation systems are not yet firmly established (Cluydts et al., 2002). There seems to be a lack of studies in order to clearly demonstrate the validity of simulators in terms of both construct (Jongen et al., 2016) and concurrent validity with respect to on-road testing (Crizzle et al., 2012; Nichols et al., 2012). Other reviews highlighted the lack of data supporting ecological (e.g., Hsieh et al., 2009; Jacobs et al., 2017; Classen et al., 2019) as well as absolute validity (i.e., the absence of significant statistical differences between effects measured on the same scale but with different tools; Kaptein et al., 1996; Classen et al., 2019). Lew et al. (2009) pointed out the lack of evidences related to test–retest reliability and the need for establishing operating characteristics of driving simulation testing (sensibility, specificity, accuracy) for specific populations. Following Shechtman (2010), one of the reasons for these issues stands in the lack of agreement about terminology used to define the concept of validity. Indeed, such terminology primarily comes from technical discipline such as engineering and computer science, but driving simulators are widespread and employed in many others scientific fields (i.e., medicine, psychology, etc.). Other works reported the need for a consensus on (a) a common set of parameters/indicators to be included in a simulator (Kraft et al., 2010), (b) settings and assessment methods of driving skills (Schreier et al., 2018), and (c) hardware (i.e., equipment) and software (i.e., scenarios) of driving simulators (Iwata et al., 2018). The huge variability on the aforementioned features hampers the comparability between simulators and makes that every research team goes on with its own device and protocol (Iwata et al., 2018). The lack of validation studies also limits the use of simulators as a tool for rehabilitation and training of driving skills. Indeed, few studies have tried to demonstrate the efficacy of driving simulation systems as a learning tool. Results seems to be inconclusive and heterogeneous and cannot be employed in order to produce a clear statement pro or versus the use of training programs based on driving simulation (Martín-delosReyes et al., 2019). In a review on rehabilitation of driving skills, it is unclear whether a driving simulation training may restore, maintain, and ensure transferability of such skills to real-world driving, and it is also unclear whether it could produce better results with respect to classical neurocognitive rehabilitation (Devos et al., 2015).

Regarding the second question, it is possible to conclude that a clear association or prediction of driving simulation performance with respect to standardized neuropsychological testing performance is present. Driving simulators thus offer the possibility to assess the same cognitive domains involved in the evaluation of fitness to drive and usually measured throughout laboratory tests, within a more ecological sensory environment. However, also for this question, the same limitations addressed for the previous one can be put forward. The lack of both validation studies and consensus on the features, parameters, and administration settings makes it difficult to collect normative data to be used for clinical evaluation of fitness to drive. Schreier et al. (2018) proposed to use both simulators and neuropsychological tools to evaluate fitness to drive in order to minimize the biases of both methods. Also, in this case, the need to validate both neuropsychological and driving simulation tools with respect to real-world driving and to standardize them for age, gender, and specific medical conditions emerges (Engleman and Joffe, 1999; Klöppel, 2009).

The third question is the less represented in the review analyzed; indeed, only for 20% of the reviews it was possible to retrieve an answer, with a substantial equality between the three categories of answer. The fidelity of driving simulation tools refers to the extent to which they simulate real-world driving experience (Kaptein et al., 1996; de Winter et al., 2007). A low-fidelity driving simulator includes a desktop and a basic equipment for simulated vehicle control, while a high-fidelity simulator usually has a 360° visual field projected on multiple monitors, a complete cockpit of an actual vehicle and a motion-based board providing kinesthetic feedback (Kaptein et al., 1996). Following Wynne et al. (2019), also for the concept of fidelity, there are issues related to the terminology and to the classification of simulators based on fidelity level with respect to on-road driving. For example, they pointed out that some research teams used the term “physical validity” to describe the fidelity, or that the lack of a common set of standard for the evaluation of fidelity usually results in three levels of classification (i.e., high, medium, and low), but there are no clear and standardized rules in order to describe the exact features for each level. In a recent review, Murray (2017) claimed that the lack of a standard device for the assessment of driving skills in individuals coming from special population (e.g., suffering from sleep disorders; Lucidi et al., 2006, 2013) may depend on the fact that driving simulators are developed and built for other considerations of driving safety than those requested for the assessment of specific population. However, the critical issue most frequently linked to fidelity is motion sickness or simulator sickness; that is, all the physiological reactions in the form of headache, nausea, and vomiting (Pinto et al., 2008). Tolerability of simulated driving experience is a fundamental issue especially in older persons, who frequently experience simulator sickness. Following Brown and Ott (2004), there are no driving simulators tailored for older people. A low performance in such people might reflect adaptation difficulties rather than deficit in driving skills *per se*. Simulation sickness seems to be the biggest issue related to simulator fidelity since it has a significant impact on both quality of measurement and drop-out rate (Malis-Gagnon et al., 2012; Iwata et al., 2018; Schreier et al., 2018). Marcotte and Scott (2004) claimed that sickness is directly related to the degree of realism. Indeed, simulators can vary in terms of visual and auditory inputs and in complexity of simulated scenario, although a relevant issue is due to the fact that only few studies provide a detailed description of the scenario, and thus it is difficult to replicate studies and generalize the results (Irwin et al., 2017). Another recurring issue is related to the risk perception in virtual reality. Even though participants carry out the task with the utmost accuracy, they are often fully aware that a collision in simulated scenario will not result in any harm and, consequently, they could not drive with the same caution they would in the real world (Marcotte and Scott, 2004). This issue starts from fidelity of driving experience but has an impact on ecological validity of measures collected with the driving simulator.

Fourth, a relevant point which emerged from the review of reviews is the difficulty in conducting a meta-analysis in order to provide a quantitative synthesis of causal relationships, predictive ability, and/or correlation between a) cognitive variables and driving simulation performance and b) driving simulation performance and on-road test (Wynne et al., 2019). Such difficulty might be given by different sources of huge heterogeneity among studies, namely, a) the availability on the market of several types of driving simulators, the large variability in the measures taken, as well as in their fidelity and reliability, b) the variability of tools and neuropsychological batteries used to measure cognitive abilities related to driving skills, and c) the variability due to experimental designs and manipulations.

There are two other considerations that might be taken into account when results from simulated driving performance are evaluated. The first one regards the distinction between predictive validity of simulated performance with respect to on-road performance or with respect to crash and collision rates (Lucidi et al., 2014, 2019; Mallia et al., 2015; Spano et al., 2019). Neither simulated driving nor on-road testing seems to be predictive of future accidents (Man-Son-Hing et al., 2007; Drazkowski and Sirven, 2011; Gupta et al., 2017; Baiardi and Mondini, 2019), and, despite the latter is considered the gold standard for assessing fitness to drive, there are few studies which investigated which measures in simulated driving might be useful to predict the risk of collision (George, 2003; Drazkowski and Sirven, 2011; Piersma et al., 2016). Since the vast majority of research on simulated driving revolves around the topic of driving safety in a preventive perspective, it would be useful to direct research efforts to find and validate measures with high predictive validity with respect to crash and collision rates in real-world driving.

The second consideration concerns the distinction between tests of *typical* and *maximum* performance. All the methods used for assessing fitness to drive, that is, neuropsychological testing, driving simulation, and on-road testing, are tests of maximum performance, requiring the individuals to exert as much effort as possible and to obtain the best performance one can do. The real-world everyday driving activity can be instead considered a test of typical performance, requiring the individual to exert an effort enough not to incur in collisions or in major violations (Lucidi et al., 2010). Such discrepancy might be one of the reasons why all the aforementioned methods are not fully adequate to capture the variability of everyday driving. The issue here is not in the specific method used for assessing but resides in a substantial difference between the behavior elicited in these two frameworks. A possible remediation in order to get a typical evaluation of fitness to drive has been developed in multicenter longitudinal studies promoted by the AAA Foundation for Traffic Safety, namely, “The longroad study–Longitudinal research on aging drivers” (Li et al., 2017), and in another project called “The Ozcandrive Project” (Marshall et al., 2013). In these projects, in-vehicle recording devices together with a GPS system were applied within the vehicle in order to collect data from everyday driving activity (i.e., position, time of the day, speed, acceleration, safety distance, lane deviation, etc.) in real time and for a prolonged period of weeks or months.

## Conclusion

In light of the results obtained and discussed above, some concluding remarks may be outlined.

First, driving simulation studies and reviews represent an increasingly relevant topic in the scientific literature on driving, especially in recent years and thanks to the technological innovations as well as to the increased computing power of hardware and software (e.g., Cipresso et al., 2018).

Second, it seems that driving simulation is a cross-cutting topic, present and widespread among different disciplines. It is also addressed with several approaches in virtue of a versatile methodology which allows the study of different aspects of driving simulation (e.g., from a human factor, medical, psychological, engineering-technical perspective).

Third, it is thus possible to observe a lack of shared and standardized methodologies and protocols, as well as the lack of a common language in the research field employing a driving simulation procedure (Wynne et al., 2019). All those factors act against the possibility to summarize findings from studies which investigate a similar relationship between driving-related variables, as well as to clearly compare driving simulation performance with other methods in order to assess fitness to drive in normal and special populations. Nonetheless, there are several evidences for considering driving simulation as a valid alternative to neuropsychological testing as well as to on-road testing for the assessment of fitness to drive.

Fourth, data coming from driving simulation studies are limited in providing generalizable results. Heterogeneity in simulators’ types, settings, driving tasks, scenarios, specific populations, and research methodologies hampers the spread of driving simulation in clinical contexts; thus, content validity is limited for specific simulators, tasks, and populations (Kraft et al., 2010; Shechtman, 2010; Verster et al., 2011; Iwata et al., 2018). Another issue related to generalizability of results comes from the fact that several studies did not report all the data captured from the software within the simulator (Irwin et al., 2017), and this makes it difficult to establish a set of measure and consequently of normative data (Kraft et al., 2010). Moreover, simulators that warrant a complete and naturalistic assessment of driving skills are expensive, cumbersome, and hardly available (Murray, 2017; Rizzo et al., 2018). Following Schreier et al. (2018), it would be useful to conduct studies aimed at both validating the same measures with different simulators and identifying the most comparable ones.

Lastly, further research efforts could be aimed at establishing a consensus statement for protocols regarding the assessment of driving behavior and fitness to drive in order to (a) use standardized cognitive and neuropsychological tests and batteries, (b) assess and compare systematically driving simulators with regard to what they measure and to their validity and fidelity, and (c) employ shared research designs and criteria for conducting studies in a given subtopic, e.g., with special populations.

The present study has three main strengths. First, it deals with a scientometric analysis on driving simulation considering the entire population of secondary studies on that topic. Two different scientific databases were analyzed since we were aware that there could have been a reduced share of overlapping between them and we wanted to reduce the risk to exclude relevant literature. The aim was thus not to carry out a comparative analysis between databases, but an exhaustive one. Indeed, there were 228 documents classified as reviews in Scopus and 151 in WoS. The final sample was composed of 298 records. This means that there were 81 duplicate records that were present in both databases, with a consequent overlap share of about 27%. This also means that, using only one database, 70 unique records using Scopus and 147 unique records using WoS would have been excluded.

Second, as far as we know, a second-order scientometric analysis including only secondary studies has never been conducted before. The rationale of a scientometric analysis on reviews lies in the fact that the authors of primary and secondary studies do not necessarily coincide. The authors of a review may not necessarily be experts on the main topic (here, driving simulation), but they may be experts on associated topics interested in undertaking an applied study using driving simulators. Moreover, it also allowed for the comparison with the recent scientometric analysis by Guo et al. (2019) on primary studies.

Third, the present study proposes a new approach integrating scientometric analysis with a review of reviews. The latter explicitly addressed the issues of the validity of simulators with respect to the gold standard for assessing fitness to drive, which remains the on-road test. Moreover, it explicitly compares the effectiveness of simulators in replacing the neuropsychological and psychometric tests frequently used in daily practice to predict driving success in special populations. This triangulation brought out two clusters of research questions, obtaining results of interest for those who intend to undertake research or are interested in proposing to stakeholders to integrate the on-road test with driving simulator assessment. Road safety professionals can rely on data providing suggestions on how simulators preach on-road tests on the one hand and how they provide suitable experimental control over the neuropsychological tests on the other, thus giving useful indications on the neuropsychological and psychometric prerequisites for fitness to drive.

The present study has some limitations. The first one comes from an issue which is always present in review and meta-analytic studies, and it is reasonable to be also present in scientometric investigations, namely, the exclusion from the analysis of the white papers and gray literature. Such literature is usually not indexed and available in official databases and can provide a relevant source of information for disseminating studies reporting null or negative results that might not otherwise be disseminated (e.g., Paez, 2017). Currently, there are no methods to assess the impact of white papers and gray literature on the results of a scientometric analysis, unlike meta-analysis for which specific techniques have been developed. In this view, results from a scientometric analysis can be biased, especially toward positive results, and the conclusions may not be fully generalizable and need to be taken with caution.

The second limitation is due to the time coverage of the literature search. Indeed, the search did not include the second half of the 2019; this could have had an impact, albeit modest, on the last time point of Figure 1 and on the number of the reviews included both in the scientometric analysis and in the review of reviews. For the sake of clarity, a new search was conducted on both Scopus and WoS on March 30, 2020, with the same search expression and produced the following results: Scopus yielded 238 reviews, with two more reviews in 2019 than those included in the data, and WoS yielded 164 reviews, with nine more reviews in 2019 than those included in the data. The two more reviews present on Scopus were also present on WoS, so in total, nine reviews were missed in 2019.

In conclusion, the present study represents an opportunity for broad-based methodological suggestions on a series of ideas: (a) heterogeneity of sources. It is typical for applied topics such as driving simulation. Indeed, this topic attracts the attention of scholars from very different disciplines. In addition to those largely expected such as engineers and computer scientists, with an ergonomics-oriented look, there can be found a wide range of data from medicine and allied disciplines such as neurosciences and psychology, each of these with different publication impacts and citational traditions. Such differentiation supports the need to derive the sources of analysis from multiple databases; (b) scarce bibliometric overlap between primary and secondary items and therefore the usefulness in some areas of conducting a second-order scientometric analysis. The widespread attention of several disciplines increases the variability of topics covered by the reviews, partially differentiating bibliometric characteristics (i.e., Authors, Institutes, Journals, Countries) of primary and secondary studies; and (c) usefulness to conduct a scientometric analysis together with a literature review, with the aim of providing a comprehensive picture of the topic by adopting two well-differentiated perspectives of analysis, which can be considered allied and complementary. The present study could be a good example of this broad-range approach.

## Author Contributions

AC, LT, ALo, and AB contributed to idea conception, data extraction, and analysis. AC, LT, ALo, AB, GS, ALi, YM, IG, and FS contributed to writing the first draft of the article. All authors contributed to article revision and approval of the final version.

## Conflict of Interest

The authors declare that the research was conducted in the absence of any commercial or financial relationships that could be construed as a potential conflict of interest.
